# Assistive technologies for diabetes care: a personalized software for progressive foot and ankle home exercises

**DOI:** 10.1186/1758-5996-7-S1-A254

**Published:** 2015-11-11

**Authors:** Cristina Dallemole Sartor, Jane Suelen Silva Pires Ferreira, Cristina Correa Oliveira, Daniel de Azevedo Figueiredo, Rafael Cruz, Isabel de Camargo Neves Sacco

**Affiliations:** 1 Universidade de Sao Paulo/Universidade Federal de Sao Paulo, São Paulo, Brazil

## Background

Assistive technologies for DM management and monitoring are powerful tools for motivation and adherence to the desirable treatment and lifestyle[1]. Losses in foot and ankle due to diabetic polyneuropathy (DPN) require a continuous care since the disease diagnosis, especially to maintain function and mobility and avoid later consequences such as ulcers[2].

## Objective

To develop a free software that personalizes a foot and ankle routine of exercises according to the patients' individual improvements. Materials and methods: Webapp system which uses Responsive Design, developed in PHP, in English or Portuguese. It was designed for Web on-line or off-line access, with applications also for smartphones (Android or iPhone) that integrates the progression of the exercises with the software. It was designed for users that do not have high speed connection with internet or have variations of the speed.

## Results

The software – SAEDD[3]-presents sessions about: a) DM and foot care recommendations; b) Foot self-assessment: the patient can choose from a list of foot problems (blister, tissue discontinuity, crack, callus, toes deformities-claw, hammer or hallux valgus-liquid, loose or fallen nail, bleeding, pus, and skin color-black, red or white) and drag and place a marker over 5 different views of the foot; c) Foot and ankle exercises: each person starts the training according to a list of selected exercises, which are described in a step-by-step video, photo and written information. A progression of approximately 8 levels of difficulty are available for each exercise that also differs in number of series and repetitions, body positions and materials used. After performing each exercise, patients have to score its effort in a visual 0-10 analogic scale. Every 5 days of training, an algorithm adjusts the progression level of the next exercises series, depending on the effort score given. For visual feedback of each exercise, a graph presents the percentages of the finished and the remaining levels.

## Conclusion

SAEDD can be recommended by health care providers to facilitate self-monitoring and promote home-based exercises. It helps the patient to be independent in the treatment, and has the main benefit of progressing according to the own patient's possibilities, which is a closer situation to a supervised therapy.
